# Two Case Presentations Infected by *Trichosporon asahii* and Treated with Voriconazole Successfully

**DOI:** 10.1155/2015/651315

**Published:** 2015-08-13

**Authors:** Hikmet Gulsah Tanyildiz, Sule Yesil, Sule Toprak, Mehmet Onur Candir, Gurses Sahin

**Affiliations:** Dr. Sami Ulus Maternity and Children's Health and Diseases Training and Research Hospital, Department of Pediatric Oncology and Haematology, Ankara, Turkey

## Abstract

*Background*. *Trichosporon asahii *is an opportunistic fungus that causes infections in immunosuppressed patients. Neutropenia developing due to malignancies is an important risk factor for fungal infection. *Case Report. *We present two pediatric oncology cases successfully treated with voriconazole after *T. asahii *infection that is known to cause fatal sepsis and invasive fungal infection. *Conclusion.* There is no conclusive evidence that the antifungal agent voriconazole is effective in the neutropenic patients infected with *Trichosporon asahii*. Liposomal amphotericin B has also been reported to be inadequate for treatment. We believe that our patients were successfully treated and survived because the antifungal agents were started early and properly, although the infection can be fatal in up to 80% of cases despite treatment.

## 1. Introduction 

*Trichosporon asahii* is an opportunistic fungal infection that is rarely seen in children and also an emerging cause of systemic fungal infection in immunocompromised hosts. It has a high mortality rate even with amphotericin B treatment [1]. Mortality from the infection can be prevented with early diagnosis and starting proper antifungal therapy at an early stage.

Although various antifungal agents have been used for treatment, the best responses were obtained with the single or combined use of liposomal amphotericin B and voriconazole [2, 3].

We present the successful treatment* T. asahii* fungemia with voriconazole in two different cases diagnosed with LCH (Langerhans cell histiocytosis) and AML (acute myeloid leukemia).

## 2. Case 1

A 2-year-old male patient was referred to our clinic for skin rash, anemia, thrombocytopenia (hemoglobin: 7.7 gr/dL, platelets: 46,000/mm^3^), and hepatosplenomegaly. Bone marrow aspiration revealed increased histiocytic activation. The result of the biopsy from the skin lesions was reported as CD1 a, CD 68, and S- 100+ Langerhans cell histiocytosis. The LCH 1994 chemotherapy protocol was started. Cladribine and cyclosporin A were added to the patient who was resistant to standard treatment. There was a prolonged febrile neutropenia episode after the second course of cladribine treatment in the patient scheduled for allogenic stem cell transplantation. The absolute neutrophil count was 200/mm^3^ and the fever 38°C on the 14th day of chemotherapy. Empiric antibiotherapy was started with meropenem, aminoglycosides, and teicoplanin. Liposomal amphotericin B (5 mg/kg/day) was added when fungemia was suspected due to the continuation of fever on the third day. No pathology was found on abdominal USG and thoracic CT imagination. There was no growth on blood cultures sent from both the lumen and the periphery of the Hickman catheter. Ciprofloxacin was added to the antibiotic treatment on the 7th day of neutropenic fever as an empirical treatment. No growth occurred until the ninth day of neutropenic fever and the 6th day of amphotericin B treatment in any of the cultures previously obtained at different times. Growth was reported in the blood culture for the first time on the 10th day of neutropenic fever and the factor was found to be* T. asahii*. Voriconazole (4 mg/kg, 2 doses) was added to the liposomal amphotericin B treatment that had been started empirically due to the continuation of the fungemia clinical picture.* In vitro* susceptibilities of opportunistic* T. asahii* fungi were determined by NCCLS M27-A2 broth in dilution MIC methods. Ciprofloxacin and teicoplanin which were started empirically had been stopped after proof of fungal infection. The Hickman catheter of the patient was removed as the fever was resistant to treatment. It was noteworthy that the fever of the patient decreased, absolute neutrophil count was increased to 600 mm^3^, and no growth was present at follow-up culture on the second day of voriconazole treatment following the removal of the catheter. No growth was found in any of the five blood cultures obtained afterwards. Oral voriconazole therapy was continued and an application was made for unrelated stem cell transplantation for the primary disease.

## 3. Case 2

A 12-year-old female patient was treated with a diagnosis of chronic myeloproliferative leukemia (CML) six years ago and referred to our clinic for chemotherapy following the development of secondary AML during follow-up. The patient's general condition was poor. The blood count on admission revealed hemoglobin of 6.6 g/dL, white cell count of 75,000/mm^3^, and platelet count of 67,000/mm^3^. Uric acid (6.9 mg/dL) and LDH (4500 IU/L) elevation and the presence of abundant myeloblasts in the peripheral smear were noteworthy. Flow cytometry (CD45+, CD34+, CD11b+, CD117+, CD13+, CD33+, MPO+, and HLA DR+) was reported to be consistent with AML M1. The patient was put on the AML-BFM 2004 protocol. Dasatinib was added to the treatment when the cytogenetic analysis showed t(9,22) positivity. The patient had neutropenic fever (absolute neutrophil count 100/mm^3^) on the 6th day after the first cycle and was started on meropenem and teicoplanin. The fever continued on the third day. Liver and kidney function test results were as follows: AST: 35 U/L (0–40 U/L), ALT: 24 U/L (0–40 U/L), and BUN: 8 mg/dL (0–23 mg/dL). Abdominal USG evaluation was conducted with a suspicion of typhlitis in the patient who had a creatinine value of 0.5 mg/dL (0.3–1.2 mg/dL) when the abdominal pain started. No pathology was seen on abdominal USG and thoracic CT imagination. Teicoplanin was stopped and vancomycin and metronidazole were added to the treatment. Liposomal amphotericin B was started as antifungal treatment on the 5th day of neutropenic fever. Growth consistent with* T. asahii* occurred in the blood culture repeated on the 5th day following three negative blood cultures and voriconazole was added to the antifungal therapy. Voriconazole had the most sensitive MIC value (0.06 *µ*g/mL) on antibiogram and the treatment was therefore continued with this medication. The fever was controlled on the 3rd day of the voriconazole treatment and the absolute neutrophil count was seen to increase to 500/mm^3^. Three blood cultures sent after voriconazole was started were found to be negative. No appropriate related donor could be found for the patient whose chemotherapy continued with a diagnosis of AML and she was scheduled to receive voriconazole treatment during chemotherapy.

## 4. Discussion

*Trichosporon* species are the second most frequently isolated yeast species after* Candida* species from cancer patients. They are found in the normal flora of the skin and respiratory tract and can cause superficial and deep fungal infections. Risk factors for fungemia due to invasive* Trichosporon* infections include hematological malignancies, neutropenia, quinolone or itraconazole prophylaxis, organ transplantation, central venous catheter (CVC) use, extensive burns, corticosteroid use, and peritoneal dialysis. Increased white blood cell count in neutropenic patients is a favorable prognostic factor for controlling this infection [1–5]. The most commonly isolated* Trichosporon* species is reported to be* T. asahii* [3].

*T. asahii* infection is usually fatal despite proper treatment. The fungus can be isolated from blood or urine cultures or postmortem tissue culture but it is usually not possible to confirm the infection at the early stage. Early diagnosis and initiating proper empirical treatment properly is therefore very important in terms of reducing mortality [6, 7]. Although various antifungal agents have been used for treatment, the best responses were obtained with the single or combined use of liposomal amphotericin B and voriconazole [3]. However, fatal pediatric cases due to* T. asahii* infection despite treatment with amphotericin B and voriconazole have been reported in the literature. A pediatric ALL case reported by Thibeault et al. died due to a* T. asahii* infection emerging during the neutropenic fever period despite treatment with the amphotericin B and voriconazole combination [1].* T. asahii* can be confused with other* Candida* species during laboratory investigations and this can be misleading regarding choosing and quickly starting the proper antifungal. Various results regarding the efficacy of amphotericin B have been reported in the literature [8]. For example, successful results with amphotericin in disseminated* T. asahii* infection were reported in some newborn cases [9]. However, susceptibility of the agent to amphotericin B can be variable and resistance can also develop [7]. Resistance develops in the* in vivo* environment with a biofilm layer produced by the* Trichosporon* species. The fungus may therefore seem to be sensitive to amphotericin B in the* in vitro* environment but the expected response may not be observed in the* in vivo* environment. The azole group antifungals have been shown to be more effective against* T. asahii* species than amphotericin B in* in vitro* studies [6–8]. As mentioned in the ESCMID guidelines, voriconazole seems to be especially effective in the triazole group in the* in vitro *environment and cases with favorable treatment results have been reported [2, 10]. There are only a few studies comparing amphotericin B and azole group agents and there is no clear approach to the treatment. The antibiotic sensitivity of the agent, the immune system, and the neutrophil count of the patient play important roles in antifungal treatment success [11].

The control of our patients' disease from the hematological point of view and the early increase in neutrophil count affected the antifungal treatment process positively. We started empirical liposomal amphotericin B therapy at the first stage without evidence of fungal infection due to the prolonged fever as we know that the disease could be fatal. However, when* T. asahii* grew in blood culture later on, we added voriconazole to the treatment until the results of antifungal susceptibility tests were available. Voriconazole had the best antifungal sensitivity susceptibility result and we therefore continued with a single agent (MIC value was 0.03 *µ*g/mL in the first case and 0.06 *µ*g/mL in the second case) (Table 1).

## 5. Conclusion

We believe that our patients were successfully treated and survived because the antifungal agents were started early and properly, although the infection can be fatal in up to 80% of cases despite treatment.

## Figures and Tables

**Table 1:** MIC values against antifungal agents of the first and second cases with *T. asahii *infection.

Antifungal agents	MIC_1_ (*µ*g/mL)	MIC_2_ (*µ*g/mL)
Amphotericin B	1	1
Fluconazole	0.50	2
Itraconazole	0.125	0.25
Anidulafungin	2	3
Posaconazole	0.25	0.5
Caspofungin	4	3
Voriconazole	0.03	0.06

MIC_1_: MIC value of the first case.

MIC_2_: MIC value of the second case.
